# Sustainable fabrication of functionally graded material type Ag/silicone rubber nanocomposite by sonochemical effects

**DOI:** 10.1016/j.ultsonch.2025.107496

**Published:** 2025-08-05

**Authors:** Yamato Hayashi, Madoka Yoshikawa, Tatsuya Shishido, Aya Kudo, Hirotsugu Takizawa

**Affiliations:** Graduate School of Engineering, Department of Applied Chemistry, Tohoku University, 6-6 Aoba, Aramaki, Aobaku, Sendai 980-8579, Japan

**Keywords:** Functionally graded materials, Composite materials, Ultrasonic, Sustainable development goals

## Abstract

In this study, a composite material consisting of silicone rubber and silver (Ag) nanoparticles was fabricated using a sonochemical approach. By irradiating silver oxide (Ag_2_O) and silicone rubber in ethanol, a functionally graded material (FGM) was synthesized, in which Ag nanoparticles were incorporated on the surface and interior of the silicone rubber. The structure of the FGM had a smaller composition of Ag nanoparticles from the surface to the inside of the silicone rubber, which changed continuously. This compositional gradient improved the heat resistance of silicone rubber by about 30°C. Although this gradient material had an extremely small Ag loading amount of about 0.5 mg/cm per unit area of silicone rubber, this material exhibited good conductivity (under 10^-4^ Ω･cm). Notably, this material can be easily fabricated using ultrasonic irradiation. Due to cavitation effects, even at room temperature and atmospheric pressure, chemical reactions for this FGM between the solvents and solutes occurred. This reaction requires only Ag_2_O, silicone rubber, and ethanol as raw materials, and the only byproduct is O_2_. Therefore, this sonochemical process is simple and sustainable, as the product can be obtained cost effectively and environmentally friendly.

## Graphical abstract

**Figure:**
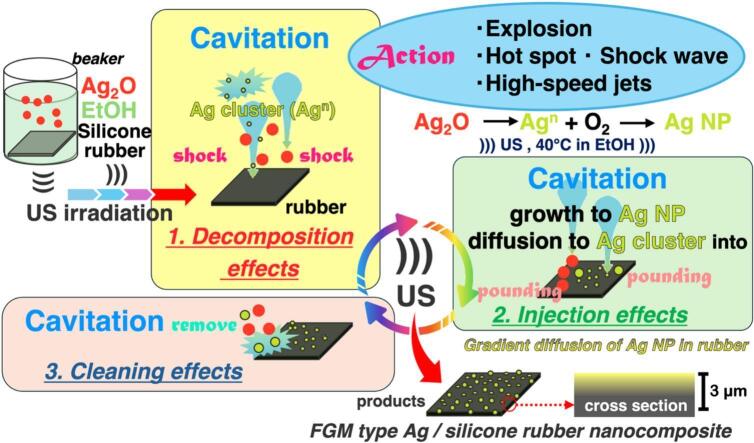

## 1 Introduction

In recent years, nanoscale compositions have attracted considerable attention in the field of material science owing to their unique physical properties[[1], [2], [3]]. The properties of organic–inorganic nanocomposites depend on the interfacial interactions between the two phases [4]. Polymer-based composite materials can enhance various physical properties by the incorporation of nanofillers, and many materials have been studied as nanofillers [[5], [6], [7]].

Various different methods enable the synthesis of nanocomposite materials [8,9], but some are complex because they involve vacuum systems or toxic chemicals [[10], [11], [12], [13], [14]]. Furthermore, these products increase the hardness of the material, leading to loss of flexibility [15], which is an inherent advantage of silicone rubber. Therefore, a more convenient method is required to compose materials to obtain flexible silicone rubber.

Ag, which is used as a nanofiller, has the highest electrical and thermal conductivities of any metal at room temperature. Rubber is typically a soft material; hence, it is expected to provide the functionality of nanofillers while retaining its flexibility. A combination of these materials offers a promising route to fabricate multifunctional organic–inorganic nanocomposites.

In this study, Ag nanoparticles and silicone rubber were composited with gradients to exploit their properties. They are generally called functionally graded materials (FGM) [16]. The structure of FGM continuously changes in composition, which can reduce stress and improve various properties (e.g., thermal, mechanical, electrical, and magnetic features) [[17], [18], [19]]. In polymer composites, conventional layered materials exhibit delamination at the interface when subjected to force; however, FGMs can successfully overcome this problem [20,21]. In addition, there are some materials whose properties vary depending on the amount and gradient ratio of the filler, which play a higher performance and multifunctional role than the usual materials [22]. FGMs have been extensively studied in recent years for applications in a wide range of industrial fields such as aerospace, automobile, optoelectronic devices, energy-absorbing structures, and biomedical implant industries [[23], [24], [25]]. For example, functionally graded materials composed of silicone resin and fused silica powder have been reported to exhibit superior thermal conductivity, coefficient of thermal expansion, and flexural properties compared to conventional polymer/ceramic matrix composites [20]. Various materials—such as epoxy/alumina [26], phenolic/graphite [27], nylon-11/glass [28], and styrene butadiene rubber/carbon black [29]—have been incorporated as fillers in other polymer based FGMs. Several methods commonly used to fabricate FGMs are centrifugal casting, powder metallurgy, additive manufacturing, plasma spraying, chemical and physical vapor deposition [25,[30], [31], [32]].

In this study, the FGM was fabricated using a green and simple sonochemical process [33]. When ultrasonic waves are applied to the liquid phase, bubbles (cavitation) are generated due to local negative pressure. These bubbles expand and contract repeatedly in response to ultrasonic pressure oscillations. Subsequently, they collapse and generate an ultrahigh-speed microjet flow [[34], [35], [36], [37]]. This collapse results in quasi-adiabatic compression inside the bubble, forming a localized reaction field, called a hotspot, with high temperatures and pressures reaching several thousand degrees Celsius and several thousand atmospheres [[38], [39], [40]]. Various chemical reactions are induced and promoted by the interaction between the hotspot and solute/solvent. This is known as the cavitation effect of ultrasonic irradiation [[41], [42], [43], [44], [45]].

We previously reported the formation of fine particles by ultrasonic irradiation [[46], [47], [48], [49]]. Especially Ag_2_O, as the raw material of Ag nanoparticles in this study, decomposes by heating at higher than 250 °C in the air. However, sonochemical processes provide a way to reduce Ag_2_O to near-room temperature. As it is a safe compound that consists of only Ag and oxygen (O), Ag_2_O is cheaper and easier to handle than other compounds. The use of ethanol (EtOH)- a safe, low cost and easily handled organic solvent, further enhances the practicality of this approach.

Based on these advantages, we fabricated Ag-rubber composites as FGMs by ultrasonic irradiation in EtOH.

## 2 Experimental

### 2.1 Composition of Ag-rubber composite

**Fig. 1:**
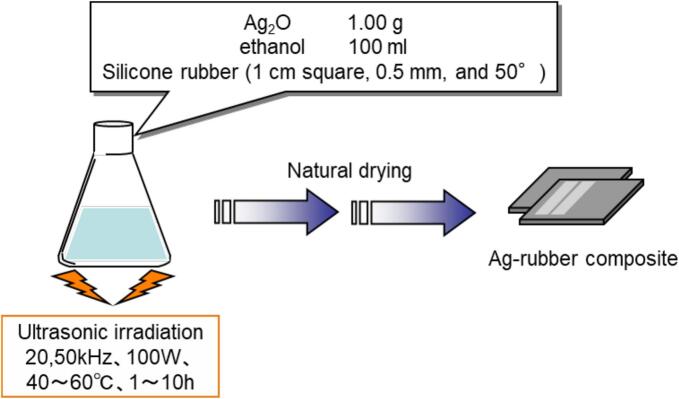
Experimental procedure of FGM type Ag-rubber nanocomposite.

Fig. 1 shows a flowchart of the composite fabrication process. Silver oxide (Ag_2_O, 1.2 g) was ball milled for 24 h, and combined with silicone rubber sheets 1 cm square, 0.5 mm thickness, and 50° hardness, and 100 ml of ethanol as a solvent was placed in a 300 mL Erlenmeyer flask. The sample was sonicated at 20 or 50 kHz, 100 W, at a temperature range of 40–60 °C. After this the samples were dried under ambient conditions. Then, partial samples were annealed in an electric furnace at 5 °C/min of heating rate and 300 °C of maximum temperature for 1 h.

### 2.2 Characterization

The X-ray diffraction (XRD) measurements was carried out with RINT-2000 diffractometer over a 2θ range of 5–90°. Field emission scanning electron microscopy (FE-SEM) images were obtained using a JSM-650 microscope. For cross sectional observations, the samples were first frozen in liquid nitrogen, split and then sputter coated with gold. Inductively coupled plasma (ICP) was recorded using an SPS-7800 instrument to determine the amount of silver loaded. Thermal properties were evaluated using a thermogravimetric differential thermal analyzer (TG-DTA) procured from TA Instruments. Finally, the electrical resistance was measured using a Resitest 8603. The resistances of selected samples were measured at elevated temperatures, i.e., 50, 100, 150, and 200 °C.

## 3 Results and discussion

### 3.1 Frequency and temperature dependence

**Fig. 2:**
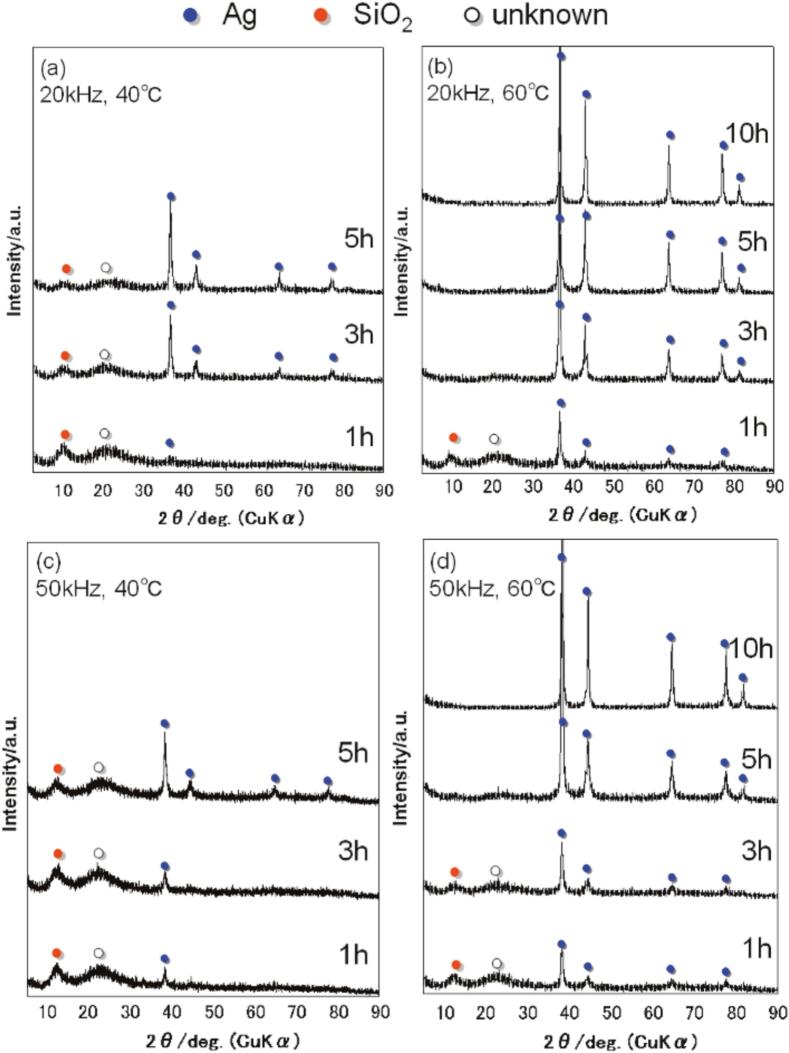
XRD patterns of surfaces on Ag-Si rubber composite under various conditions.

Fig. 2 shows the XRD patterns of the surfaces of the Ag-Si rubber composites prepared under various conditions. The peak intensity of Ag increased significantly at lower frequencies and higher temperatures, indicating that these conditions facilitate reduction of Ag_2_O. With respect to ultrasonic irradiation, the lower the frequencies, the stronger the bubbles generated, while the higher the temperature, the greater the vapor pressure of the liquid phase, thereby enhancing the bubble generation frequency [42,50]. Under high-temperature conditions, the cavitation becomes weaker compared to low-temperature conditions [38]. However, in the reduction of Ag_2_O, which proceeds easily, cavitation can be highly effective if the number of cavitations is increased, and many reaction fields are created. Therefore, lower frequency and higher temperature conditions are considered to promote reduction and increase the number of Ag nanoparticles, which in turn increases the adhesion of Ag nanoparticles to the silicone rubber surface. There was no correlation between the amount of reduced Ag and the granular variation (Fig. S1). The average particle sizes were approximately 35–45 nm at 20 kHz and 25–40 nm at 50 kHz, with slightly smaller particles at 50 kHz.

### 3.2 Measurement for the weight of composited Ag particles

**Fig. 3:**
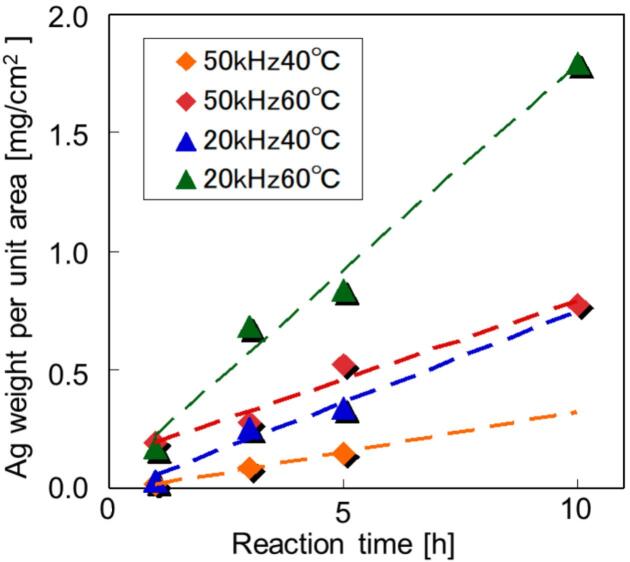
Results of determination for composite Ag by ICP.

Fig. 3 shows the composite Ag weight per unit area of the sample for the Ag-Si rubber composite measured by ICP measurements. The amount of Ag tends to increase at higher temperatures and lower frequencies. Under each condition, the amount of Ag was proportional to the irradiation time. The maximum amount of Ag was 1.796 mg/cm^2^ for samples prepared at 20 kHz, 60 °C, and 10 h (Table S1). This is consistent with the trends observed in X-ray results. Despite this, overall Ag loading remained extremely low.

### 3.3 Evaluation of heat property

**Fig. 4:**
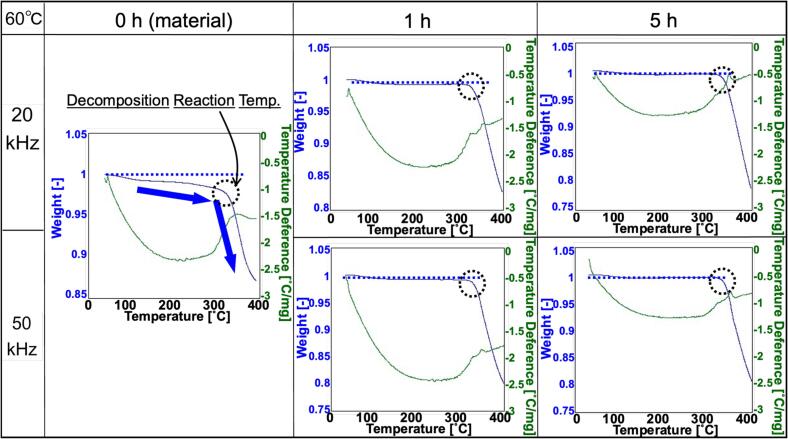
TG-DTA curves of samples sonicated at 20 kHz, 60 °C, and 100 W for 1 and 5 h.

Fig. 4 shows the TG-DTA curves of the samples sonicated at 20 kHz, 60 °C, and 100 W for 1 h and 5 h. The blue and green lines in the figure represents thermogravimetric (TG) curves and differential thermal (DT) curves, respectively. The weight of silicone rubber as a single decreased slowly with increasing temperature up to around 300 °C, and decomposition with rapid weight loss occurred at 320 °C. On the other hand, the Ag-composites (20, 50 kHz, 60 °C, and 1, 5 h irradiation) showed no weight loss and decomposition temperatures of 330–350 °C, up to 30 °C higher than that of silicone rubber. Thus, it is clear that the thermophysical properties were improved by forming a composite of Ag with crude silicone rubber. The DT curve indicating the endothermic reaction was smaller for the sample irradiated for 5 h than for the one irradiated for 1 h. This change can be attributed to the increased amount of silver supported on the rubber matrix due to prolonged irradiation, which in turn leads to a relative decrease in the rubber content in the composite.

### 3.4 Measurement and evaluation of the specific resistance value

**Fig. 5:**
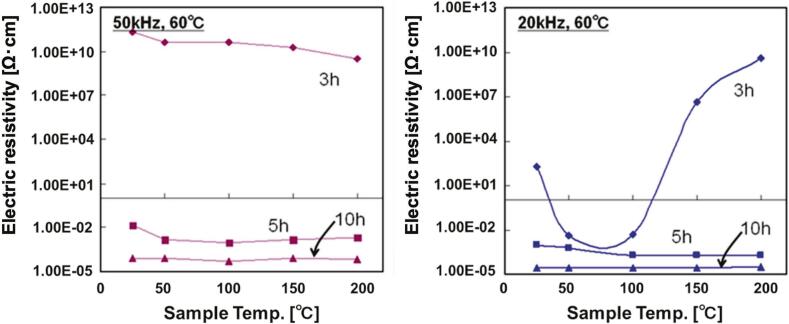
Electric resistivity measurements of samples prepared at 50 kHz, 60 °C and 20 kHz, 60 °C.

Fig. 5 shows the results of electrical resistance measurements while heating Ag composite samples prepared under 20, 50 kHz, and 60 °C conditions. The specific resistance of the silicone rubber material was 5.65 × 10^10^ Ω･cm, and the specific resistance was calculated assuming that the thickness of the Ag composite part of the sample was uniformly 4 μm. Samples irradiated for more than 5 h under each condition exhibited low resistivity values of 10^-3^ to 10^-4^ Ω·cm. Therefore, even a small amount of Ag nanoparticles can provide conductivity to the silicone rubber, which does not exhibit electrical conductivity. Furthermore, no significant change in resistance was observed upon heating. Since the base material, silicone rubber, has heat resistance that does not decompose or deteriorate under temperature conditions of 200 °C, it is expected to be practical as an electroconductive material with thermal stability.

The sample prepared at 50 kHz for 3 h showed almost the same resistance as the silicone rubber material, although the resistance decreased slightly with heating. Based on the ICP measurements mentioned above, the amount of Ag in this sample was 0.274 mg/cm^2^, which was lower than that of the other samples. This suggests that a conductive path on the silicone rubber could not be formed.

In the 20 kHz and 3-h condition, the sample showed a decrease in resistance when heated up to 80 °C, but a rapid increase in resistance when heated above 80 °C. This indicates that the sintering of the Ag particles caused percolation, resulting in a significant decrease in resistivity [51,52]. Furthermore, heating the sample increased its strain, and the resistance also increased because the contact area of the Ag particles decreased.

**Fig. 6:**
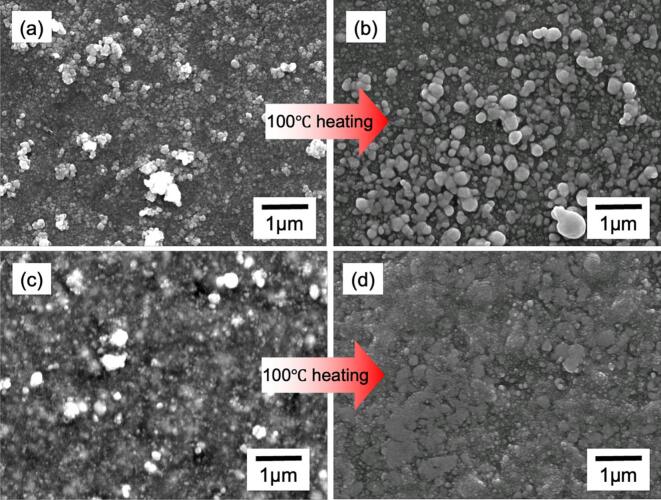
FE-SEM images for sample surfaces prepared under 20 kHz and 60 °C condition. Irradiation time of 1 h (a) before heating and (b) after heating. For 5 h (c) before heating and (d) after heating.

Fig. 6 shows the FE-SEM images of the sample surfaces before and after heat treatment at 100 °C for samples prepared by irradiation at 20 kHz for 1 and 5 h at 60 °C. The particles on the surface, the Fig. 6 (a) show particles of 0.2–0.3 μm with low existence density, Fig. 6 (b) shows agglomerated particles of larger size, and slightly higher existence density than Fig. 6 (a). Fig. 6 (d) shows that the particles appear to fuse upon heating. From the grading analysis, the average particle size of the sample without heat treatment was 25–45 nm, suggesting that the Ag particles on the surface agglomerated with neighboring particles upon heating. Silver nanoparticles are commonly stabilized with organic additives to prevent aggregation, and high temperatures (> 150 °C) are typically required to remove these additives via evaporation or decomposition during the sintering process [53]. However, in this study, it was confirmed that sintering of the particles and a reduction in resistivity can be achieved by heating at the relatively low temperature of 100 °C. This process enables sintering at low temperatures by supporting Ag nanoparticles directly on the rubber surface without the use of additives such as dispersants [53,54]. Therefore, this material can adjust its resistance according to the temperature; thus, this property might potentially be used as a temperature sensor.

From the above, it can be said that this sample is a composite material because compositing Ag with silicone rubber gave it a new function for electrical conductivity and improved its thermophysical properties, as discussed in Section 3.3 [1].

### 3.5 Sectional observation and elemental analysis

**Fig. 7:**
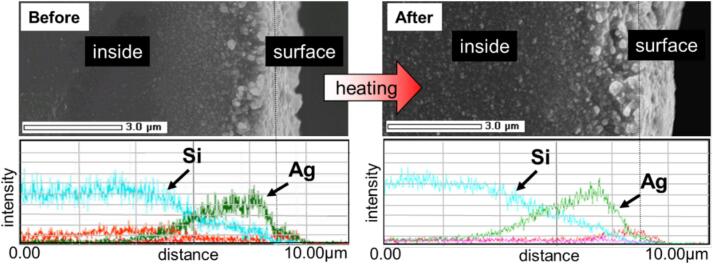
SEM images and linear analysis of the sample irradiated at 20 kHz and 60 °C for 10 h.

Fig. 7 shows sectional images of the sample with the highest Ag content at 60 °C, 20 kHz, and 10 h before and after heating to 100 °C, and the corresponding EDS line analysis results. The Ag peak decreased in intensity from the surface area to the inside, whereas the Si peak decreased in intensity from the inside to the surface. Therefore, the Ag nanoparticles and Si rubber were graded in the samples fabricated in this study. The high-temperature cavitation caused by the ultrasonic irradiation is believed to have caused the thermal motion of the Ag nanoparticles, which diffused into the silicone rubber. Generally, composite materials with two or more constituent phases, whose compositions change continuously, are called FGMs. They are expected to disperse internal stresses and provide various benefits such as improved fracture toughness and thermal properties [55].

The composite depth from the surface of this FGM was 4.7 μm before heating and 5.4 μm after heating. The specimens were compounded more deeply by heating. This graded composite and deep diffusion upon heating were also observed in the other samples. Heating can be expected to produce the thermal motion of Ag nanoparticles, which has an effect similar to cavitation, and the fillers diffuse more widely. Therefore, it is expected that Ag nanoparticles will be less prone to oxidation owing to the reduced contact with O_2_ in the air, thus improving composite stability.

### 3.6 Mechanism for Ag-rubber composite preparing

**Fig. 8:**
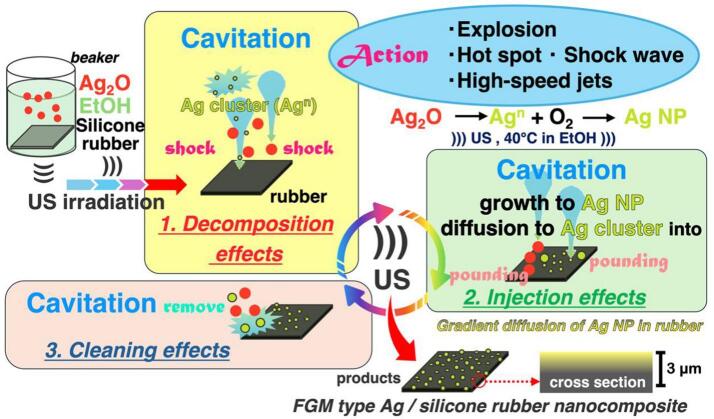
Mechanism for Ag-rubber composite with ultrasonic irradiation.

Fig. 8 shows the mechanism of Ag-rubber composite formation based on previous evaluations. The following three steps were observed when Ag_2_O and silicone rubber were added to ethanol and irradiated with ultrasound waves.

STEP1 describes the decomposition and reduction of Ag_2_O. The cavitation generated by the ultrasonic irradiation creates localized hotspots of high temperature and pressure as it collapses. Depending on the solvent and frequency condition, these temperatures reach about 5000 °C and pressure up to about 1000 atmospheres. Furthermore, on solid surfaces, cavitation collapse is accompanied by high-velocity jet flow [34,35,37,38,40]. This shock wave decomposed Ag_2_O, and Ag nanoparticles were formed. In addition to the formation of Ag nanoparticles via such decomposition, reduction by the solvent also occurs. Since ethanol is a reducing solvent, it is considered that ethanol and its radical species contribute to the reduction [46,49,56]. The Ag clusters generated through such reactions are highly unstable [49,57] and become stabilized by attaching to the silicone rubber surface. The jet flow is considered to contribute to the promotion of Ag cluster attachment at this stage.

STEP2 involved the growth and injection of Ag cluster and nanoparticles. Transient cavitation bubbles are formed under relatively low-frequency ultrasonic irradiation (20 and 50 kHz), eventually leading to their collapse [58,59]. The collapse of such cavitation bubbles generates microjets and shock waves on solid surfaces. These physical effects provide thermal and mechanical energy capable of exfoliating even graphite [60]. Polymers have atomic-sized holes called free volumes [[61], [62], [63]], which exist in silicone rubbers. Such physical effects with significant thermal and mechanical energy are considered to enlarge these free volumes and facilitate the diffusion of Ag clusters. These clusters collide with the rubber surface inside the voids, thereby initiating nucleation and promoting particle growth. Because the voids are nanosized [64,65], microsized Ag_2_O particles could not disperse from the silicone rubber surface to the interior, and only Ag nanoparticles are selectively composited. These physical effects also influence silicone rubber. However, since silicone rubber shows very high stability [66,67], the effects of polymer chain scission caused by cavitation and degradation by ethanol, are considered to be small.

In STEP 3, the excess particles are washed away. Various physical effects caused by cavitation [[68], [69], [70], [71], [72]]lead to the detachment of excessively deposited Ag nanoparticles and Ag_2_O microparticles from the silicone rubber surface. Due to the removal of coarse Ag nanoparticles, the average particle size of the deposited Ag nanoparticles decreased in the 5 h sample at 60 °C.

By repeating STEP1-STEP3, the FGM type Ag/silicone rubber composite was synthesized. As discussed above, various effects are involved in this sequence of processes, including not only ultrasonic-induced injection and diffusion, but also the promotion of chemical reactions. Such physical effects are known to become more pronounced at lower frequencies [37,73,74]. Therefore, the injection of Ag nanoparticles is promoted at lower frequencies. On the other hand, desorption of Ag nanoparticles due to cleaning effects may also increase. The reduction of Ag_2_O by ethanol proceeds more readily at elevated temperatures. In this study, it is presumed that both the injection effect at low frequency and the acceleration of the reduction reaction at high temperature contributed synergistically, resulting in the highest loading of Ag nanoparticles under the condition of 20 kHz and 60 °C.

## 4 Conclusions

In this study, nanocomposites were produced using Ag and manufactured silicone rubber, and their properties were evaluated. The Ag nanoparticles and silicone rubber were graded using ultrasonic irradiation. It was also confirmed that the conductive Ag provides electrical conductivity to the insulating silicone rubber and that the strength of the conductivity varies with the degree of heating. This allows it to be used as a sensor, which changes its resistance with temperature when used as a conductor. Furthermore, the composite sample exhibited improved heat resistance compared to silicone rubber, and these properties were demonstrated while retaining the unique flexibility of the silicone rubber. The sample prepared in this study was a graded functional nanocomposite material that combined electrical conductivity, heat resistance. Due to the extremely low filler loading, this composite material is expected to retain the inherent flexibility of the silicone rubber. Unlike conventional methods, this material can be synthesized by ultrasonication at room temperature using only Ag, silicone rubber, and ethanol, which are inexpensive and safe. This process is economical and environmentally friendly because it is simple, requires no additional reagents, and produces no waste, making it a sustainable method.

## CRediT authorship contribution statement

**Yamato Hayashi:** Writing – review & editing, Project administration, Methodology, Investigation, Funding acquisition, Conceptualization. **Madoka Yoshikawa:** Writing – original draft, Data curation. **Tatsuya Shishido:** Data curation. **Aya Kudo:** Formal analysis. **Hirotsugu Takizawa:** Supervision.

## Declaration of competing interest

The authors declare that they have no known competing financial interests or personal relationships that could have appeared to influence the work reported in this paper.
