# Supplementary material for: Sustainable fabrication of functionally graded material type Ag/silicone rubber nanocomposite by sonochemical effects
Source: Ultrason Sonochem. 2025 Aug 5;120:107496. doi: 10.1016/j.ultsonch.2025.107496 (PMC12345318; doi:10.1016/j.ultsonch.2025.107496)
Supplement: Supplementary Data 1 [file mmc1.docx]

**Supporting Information**

**Sustainable fabrication of functionally graded material type Ag/silicon rubber nanocomposite by sonochemical effects**

Yamato Hayashi, Madoka Yoshikawa, Tatsuya Shishido, Aya Kudo, Hirotsugu Takizawa

Graduate School of Engineering, Department of Applied Chemistry, Tohoku University, 6-6 Aoba, Aramaki, Aobaku, Sendai 980-8579, Japan

Corresponding author: Y. Hayashi; E-mail address: Yamato.hayashi.b6@tohoku.ac.jp


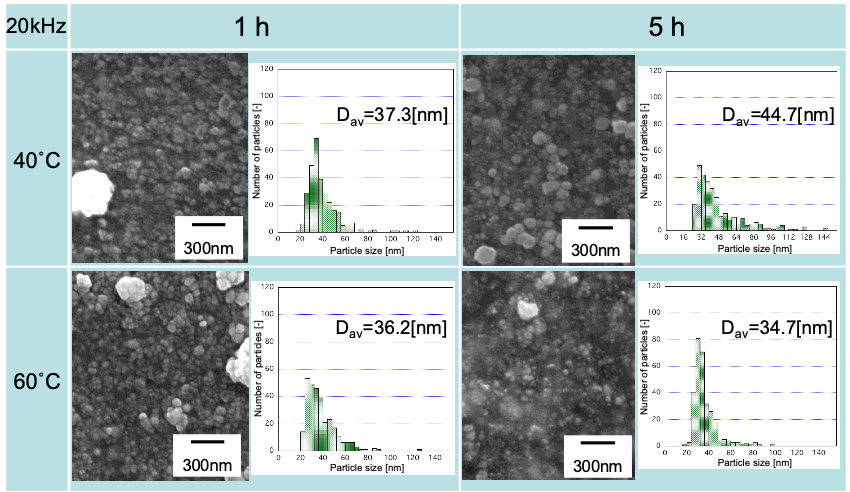


Fig.S1 (a) The SEM images and the results of the grading analysis (20 kHz)


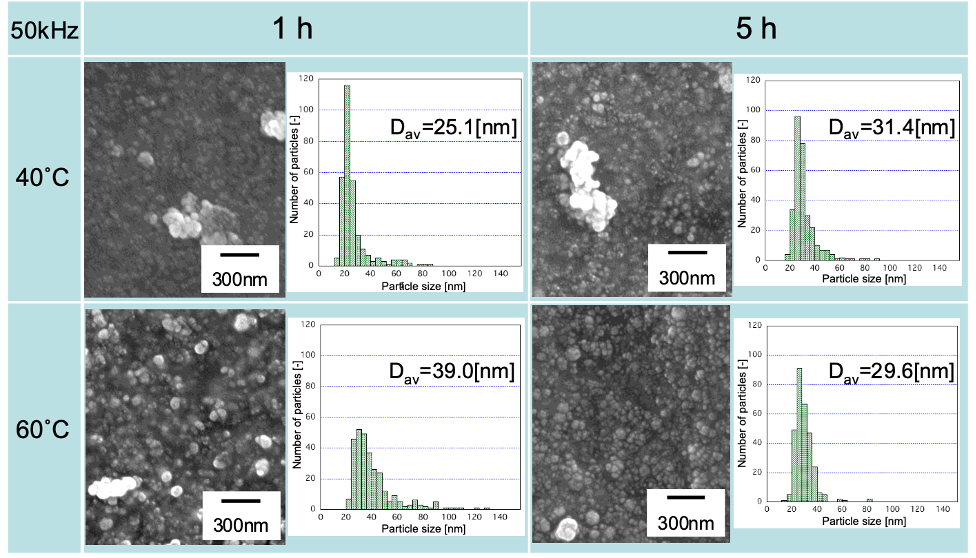


Fig.S1 (b) The SEM images and the results of the grading analysis (50 kHz)

TableS1 Ag weight per unit area

|  | | **Ag weight per unit area [mg/cm^2^]** | | | |
| --- | --- | --- | --- | --- | --- |
|  |  | 1 h | 3 h | 5 h | 10 h |
| 20 kHz | 40℃ | 0.013 | 0.087 | 0.148 | - |
|  | 60℃ | 0.192 | 0.274 | 0.519 | 0.773 |
| 50 kHz | 40℃ | 0.033 | 0.256 | 0.340 | - |
|  | 60℃ | 0.170 | 0.683 | 0.833 | 1.796 |

TableS2. a comparison of filler content and electrical performance

in conventional conductive silicone rubber composites

| **Filler** | **Filler content (wt%)** | **Electrical**  **Performance** | **Reference** |
| --- | --- | --- | --- |
| Silver-Coated Aluminum Powder | 58.3 | 1.5 × 10^4^ S/m | [1S] |
| Core-shell Ag@Al microspheres | 66.7 | 82.8 S/m | [2S] |
| silver-plated carbon fibers | 33.3 | 5 × 10^3^ S/m | [3S] |
| Ag-coated Ni particles | 47.5 | 10^-3^ Ω | [4S] |
| Sulfhydryl modified conductive carbon black/Ag hybrid filler | 10 | 10 S/m | [5S] |
| Nickel-coated graphite | 66.7 | 0.01 Ω･cm | [6S] |
| Carbon black-carbon nanotube hybrid filler | 13 | 248.80 S/m | [7S] |

[1S] Zhuang, *et al*., *J Appl Polym Sci*, **142**: e57024. (2025) https://doi.org/10.1002/app.57024

[2S] Jiahao Sun, *et al*., *J Prog Org Coat*, **207**, 109419 (2025). https://doi.org/10.1016/j.porgcoat.2025.109419

[3S] Yang Chen, *et al*., *Chin J Polym Sci* **42**, 864–873 (2024). https://doi.org/10.1007/s10118-024-3108-6

[4S] Dong Yeon Ha, *et al*., *ACS Appl. Electron. Mater.* **4**, 5809–5815 (2022). https://doi.org/10.1021/acsaelm.2c00971

[5S] Yanli Dou, *et al*., *RSC Adv.*, **12**, 1184-1193 (2022). https://doi.org/10.1039/D1RA08649A

[6S] Hua Zou, *et al*., *J. Appl. Polym. Sci.*, **115**, 2710-2717 (2010). https://doi.org/10.1002/app.29901

[7S] Pan Song, *et al*., *J. Composites B*, **191**, 107979 (2020). https://doi.org/10.1016/j.compositesb.2020.107979
